# Dysbiosis and Ecotypes of the Salivary Microbiome Associated With Inflammatory Bowel Diseases and the Assistance in Diagnosis of Diseases Using Oral Bacterial Profiles

**DOI:** 10.3389/fmicb.2018.01136

**Published:** 2018-05-30

**Authors:** Zhe Xun, Qian Zhang, Tao Xu, Ning Chen, Feng Chen

**Affiliations:** ^1^Department of Preventive Dentistry, Peking University School and Hospital of Stomatology, Beijing, China; ^2^Central Laboratory, Peking University School and Hospital of Stomatology, Beijing, China; ^3^National Engineering Laboratory for Digital and Material Technology of Stomatology, Beijing Key Laboratory of Digital Stomatology, Beijing, China; ^4^Department of Gastroenterology, Peking University People’s Hospital, Beijing, China

**Keywords:** inflammatory bowel diseases, microbiome, saliva, dysbiosis, clusters, indicator species analysis, 16S rRNA gene

## Abstract

Inflammatory bowel diseases (IBDs) are chronic, idiopathic, relapsing disorders of unclear etiology affecting millions of people worldwide. Aberrant interactions between the human microbiota and immune system in genetically susceptible populations underlie IBD pathogenesis. Despite extensive studies examining the involvement of the gut microbiota in IBD using culture-independent techniques, information is lacking regarding other human microbiome components relevant to IBD. Since accumulated knowledge has underscored the role of the oral microbiota in various systemic diseases, we hypothesized that dissonant oral microbial structure, composition, and function, and different community ecotypes are associated with IBD; and we explored potentially available oral indicators for predicting diseases. We examined the 16S rRNA V3–V4 region of salivary bacterial DNA from 54 ulcerative colitis (UC), 13 Crohn’s disease (CD), and 25 healthy individuals using Illumina sequencing. Distinctive sample clusters were driven by disease or health based on principal coordinate analysis (PCoA) of both the Operational Taxonomic Unit profile and Kyoto Encyclopedia of Genes and Genomes pathways. Comparisons of taxa abundances revealed enrichment of *Streptococcaceae* (*Streptococcus*) and *Enterobacteriaceae* in UC and *Veillonellaceae* (*Veillonella*) in CD, accompanied by depletion of *Lachnospiraceae* and *[Prevotella]* in UC and *Neisseriaceae* (*Neisseria*) and *Haemophilus* in CD, most of which have been demonstrated to exhibit the same variation tendencies in the gut of IBD patients. IBD-related oral microorganisms were associated with white blood cells, reduced basic metabolic processes, and increased biosynthesis and transport of substances facilitating oxidative stress and virulence. Furthermore, UC and CD communities showed robust sub-ecotypes that were not demographic or severity-specific, suggesting their value for future applications in precision medicine. Additionally, indicator species analysis revealed several genera indicative of UC and CD, which were confirmed in a longitudinal cohort. Collectively, this study demonstrates evident salivary dysbiosis and different ecotypes in IBD communities and provides an option for identifying at-risk populations, not only enhancing our understanding of the IBD microbiome apart from the gut but also offering a clinically useful strategy to track IBD as saliva can be sampled conveniently and non-invasively.

## Introduction

The oral cavity, which bridges the gap between exogenous substances and the internal environment, has been regarded as a valuable target for human microbial exploration (Huttenhower et al., 2012). The diverse microbiota inhabiting the mouth, which comprises over 700 prevalent taxa (Dewhirst et al., 2010), contributes significantly to maintaining the oral and extra-oral health of the host (Wade, 2013). The roles of oral microbes in mediating various diseases were first described in the 1890s by the pioneering oral microbiologist W. D. Miller, who proposed that microorganisms in the oral cavity and their products could exert a profound influence on multiple diseases, both local and general, due to dental bacteremia, which was called “oral focal infection theory” (Miller, 1891). In recent decades, the major advances of scientific techniques in terms of microbial detection, identification, and classification, especially the emergence of high-throughput next-generation sequencing (NGS) technologies (Metzker, 2010), has led to the validation and enhanced understanding of this theory. Specifically, numerous reports have elaborated the involvement of members of the commensal oral microbiota in the etiopathogenesis of systemic diseases, such as cardiovascular diseases (Figuero et al., 2011), adverse pregnancy outcomes (Han, 2011), rheumatoid arthritis (Yeoh et al., 2013), intestinal inflammation (Atarashi et al., 2017), and colorectal cancer (Nakatsu et al., 2015), among others. The entry of a sufficient load of oral opportunistic or pathogenic bacteria into the blood circulation via the oral mucosal barrier during daily oral hygiene or tooth treatment procedures could result in abnormal local and systemic immune and metabolic responses and nutrient digestion in an immunocompromised population, indicating the pathogenic basis of the oral microbiota (Homann et al., 2000; Ahn et al., 2012; Slocum et al., 2016). In turn, global immune dysregulation could also act as a direct modifier of the oral microbiota. In addition to pathophysiological roles, oral microflora are valuable biomarkers for disease monitoring or stratification due to their advantageous characteristics (Huang et al., 2014; Teng et al., 2015; Zhang et al., 2015). Oral samples are non-invasively accessible and easily processed, which enhances the efficiency of sampling and sample analysis; furthermore, studies have demonstrated that the mouth harbors a less changeable community than the gut and skin over time (Costello et al., 2009), supporting the long-term stability of oral bacterial markers.

Inflammatory bowel diseases (IBDs) represent a group of chronic relapsing inflammatory conditions of the gastrointestinal tract, which are categorized into two clinical types–ulcerative colitis (UC) and Crohn’s disease (CD) (Miyoshi and Chang, 2017). These diseases affect as many as millions of people globally, most of whom are in the prime of their life; moreover, the incidence of these conditions is rising considerably in newly industrialized areas (Molodecky et al., 2012; Kaplan and Ng, 2017). Currently, this type of life-long condition can be medically managed but not cured, and it is associated with a higher risk of colon cancer (Kaser et al., 2010). The primary symptoms of IBD relate to the intestine, mainly manifesting as persistent diarrhea, abdominal pain, and gastrointestinal bleeding (Rubin et al., 2012); however, many patients also present extra-intestinal complications, typically related to oral, skin, joint, eye, and bone lesions as well as body weight loss (Lankarani et al., 2013). Interestingly, oral lesions can sometimes predate the intestinal manifestations by months to years (Riggio et al., 1997; Sigusch, 2004). At present, the etiology of IBD is still not fully understood, but the abnormal mucosal immune response to environmental and microbial agents in genetically susceptible individuals provides information about the pathogenesis of IBD (Miyoshi and Chang, 2017). Dysbiosis of the gut microbiota is clearly correlated with IBD (Kaplan and Ng, 2017; Miyoshi and Chang, 2017), including a reduced gut biodiversity, depletion of members of the phyla Firmicutes and Bacteroidetes, and enrichment of Proteobacteria (Ott et al., 2004; Gophna et al., 2006; Frank et al., 2007; Walker et al., 2011).

Nevertheless, other members of the human microbiome that may also play roles in this condition should be investigated to acquire comprehensive knowledge of IBD; however, they are currently poorly understood. Given the noteworthy role of oral microbes in diverse systemic diseases, we hypothesized in this study that the oral microbiome has an altered structure, composition and function due to host-microbiome interactions, and we explored stratification of patients and biomarkers indicative of diseases depending on oral microbial profiles. To date, three studies have described variation in the oral microbial diversity and composition in human IBD patients (Docktor et al., 2012; Said et al., 2014; Kelsen et al., 2015). Docktor et al. (2012) reported decreased biodiversity of the tongue microbiota with a loss of Fusobacteria and Firmicutes in pediatric CD patients compared with healthy controls (HCs); conversely, Kelsen et al. (2015) showed no differences in the bacterial richness of subgingival samples from pediatric CD patients but described a significantly distinct microbial structure with increased proportions of *Capnocytophaga*, *Rothia*, and TM7. Similar to Kelsen, Said et al. (2014) discovered no alterations in microbial richness and diversity but found differences in the composition of the salivary microbiota of adult IBD patients in comparison to healthy individuals. Said et al. (2014) observed that the abundances of the phylum Bacteroidetes and genera *Prevotella* and *Veillonella* were significantly increased in both UC and CD individuals, while those of the phylum Proteobacteria and genera *Streptococcus* and *Haemophilus* were reduced in IBD patients. However, the abundances of IBD-related taxa were discrepant in these studies, possibly because their sampling locations, patient demographics, and sequencing strategies differed (Xu et al., 2015; Fouhy et al., 2016). Moreover, previous data mainly concentrated on the oral microbial structure and composition in IBD patients, but the functional changes in the oral community and roles of IBD-associated bacteria remain uninvestigated. Furthermore, little is known about the microbial variability within UC and CD communities and reliable oral indicators of these diseases.

Therefore, in the present study, we conducted a case–control analysis of the salivary microbiota to investigate (1) the dysbiosis of the oral microbiome based on structure, composition, and function; (2) whether different community ecotypes were present within UC and CD patients and if the ecotypes correlated with demographic and clinical characteristics; and (3) effective indicators of these diseases. We collected unstimulated saliva samples from Chinese adults with IBD and HCs to assess the microbial variation using 16S rRNA gene sequencing. We sampled saliva because it well represents the overall oral microbial population and can be readily obtained with minimal discomfort to patients and healthy volunteers. This study aimed to broaden our understanding of other members of the human microbiome in IBD patients besides the gut.

## Materials and Methods

### Subject Selection

All participants with IBD in this study were recruited from the Department of Gastroenterology at Peking University People’s Hospital in Beijing, China, from October 2016 to February 2017. Healthy volunteers were chosen from a population undergoing routine dental care in the Department of Preventive Dentistry at Peking University Hospital of Stomatology in Beijing, China, from December 2016 to February 2017. For the IBD patients, individuals received a diagnosis according to their clinical symptoms, physical signs, and colonoscopic, histological, and pathological examinations of the intestinal mucosa by professional physicians. IBD patients were enrolled per the following criteria: (1) age greater than 18 years; (2) no other reported systematic diseases except IBD; (3) no ingestion of probiotics or antibiotics during at least the previous 4 weeks; (4) not pregnant or lactating; and (5) no evidence of untreated or uncontrolled caries or periodontal disease, precancerous or cancerous oral lesions, and oral candidiasis after dental examination by a dentist. In total, 57 UC and 13 CD patients with either active or remissive disease were included in the study, and all of them were receiving or started to receive standardized medications at the beginning of the study. Concomitantly, 25 healthy adults were enrolled who reported no oral or systematic diseases, had not taken probiotics or antibiotics in the preceding 4 weeks and were not pregnant or lactating. All participants were instructed to avoid eating or drinking for 1 h before oral sampling.

The subjects were acquainted with the nature of the experiment and provided informed consent before participating in the study. All study procedures were approved by the Biomedical Ethics Committee at Peking University.

### Sample Collection

Every IBD patient was sampled at the time of enrollment. In addition, three UC and three CD patients with active diseases were selected for longitudinal observation, each of whom also provided two additional samples at 1 and 2 months after the baseline to identify changes in their microbiota. During this period, they underwent routine therapies. A total of 5-mL of unstimulated saliva was collected from each IBD patient and healthy control using a 50-mL sterile Eppendorf tube; each sample was then immediately placed in an ice bag using an insulating polystyrene foam container and delivered to the Central Laboratory at Peking University Hospital of Stomatology within 4 hours for processing. In the laboratory, the samples were centrifuged at 12,000 rpm for 10 min at 4°C and stored at -80°C prior to DNA extraction.

Besides, 40 UC and 13 CD patients underwent blood tests in order to monitor medication reactions on their day of salivary collection. Therefore, their blood test results (complete blood counts) were recorded along with sampling. The demographic [gender, age and body mass index (BMI)] and clinical (disease activity, duration, extracolonic symptoms) data for every subject were also recorded in this study.

### DNA Extraction, 16S rRNA Gene Library Preparation and Sequencing

Community DNA from the collected saliva samples was extracted using a TIANamp Bacteria DNA Kit (Tiangen Biotech, Beijing, China) following the manufacturer’s instructions. The DNA purity was determined based on the A260/A280 ratio using a NanoDrop 8000 Spectrophotometer (Thermo Fisher Scientific, Carlsbad, CA, United States), and the DNA integrity was verified by 0.8% agarose gel electrophoresis. A negative control containing only buffer was included during DNA extraction and quantification. The extracts were stored at -20°C before further use.

Genomic DNA was used as the template for bacterial 16S rRNA gene amplification with the barcoded primers 338F (5′-ACTCCTACGGGAGGCAGCAG-3′) and 806R (5′-GGACTACHVGGGTWTCTAAT-3′), which target the V3–V4 hypervariable region. For each sample, a barcode sequence was added to the 5′ end of the forward and reverse primers. PCR was performed on a Mastercycler Gradient (Eppendorf, Hamburg, Germany) using 50-μL reaction volumes containing 5-μL of 10 × Pyrobest Buffer, 4-μL of dNTPs (2.5 mM), 0.3-μL of Pyrobest DNA Polymerase (2.5 U/μL, TaKaRa Code: DR005A, Japan), 2-μL of template DNA (30 ng), 2-μL of each forward and reverse primer (10 μM), and 34.7-μL of ddH_2_O. Thermal cycling consisted of an initial denaturation at 95°C for 5 min, followed by 30 amplification cycles of 95°C for 45 s, 55°C for 50 s, and 72°C for 45 s, with a final extension at 72°C for 10 min. Three PCR products per sample were pooled to mitigate reaction-level PCR biases. The PCR products were purified using a QIAquick Gel Extraction Kit (QIAGEN, Hilden, Germany) and quantified using a Real-time PCR system, and the libraries were sequenced using the Illumina MiSeq platform (Illumina, San Diego, CA, United States) with 2×300-bp paired-end (PE) sequencing.

Three baseline samples from the UC patients were excluded from this analysis due to insufficient amounts of DNA, resulting in a total of 104 sequenced samples (60 samples from 54 UC patients, 19 samples from 13 CD patients, and 25 samples from 25 healthy controls).

### Sequencing Data Processing

Sequencing data were initially demultiplexed using customized Perl scripts that considered the unique barcodes assigned to each sample. The reads were then assembled using Fast Length Adjustment of Short Reads software (FLASH, ver. 1.2.11) based on the overlap of the PE reads (Magoc and Salzberg, 2011). The data were denoised by removing sequences with ambiguous base calls, <75% of consecutive high-quality base calls and <30 of average quality values using Quantitative Insights Into Microbial Ecology (QIIME, ver. 1.8.0) (Caporaso et al., 2010). To cluster the pre-processed sequences into Operational Taxonomic Units (OTUs), which are defined by the intrinsic phenotypic similarities that constitute candidate taxa (Navas-Molina et al., 2013), the closed-reference strategy for OTU picking was employed, using uclust_ref at a minimum sequence identity of 97% against the Greengenes reference database (ver. 13_8). Reads without hits in the reference database were discarded from downstream analyses. Taxonomies were further assigned according to the Greengenes database (DeSantis et al., 2006; McDonald et al., 2012).

The OTU table was used to start the downstream analysis. First, to reduce the problem of spurious OTUs, singleton OTUs were excluded. Then, 10,664 pre-processed sequences were rarefied randomly for each sample to reduce the effects of variable sequencing depths on alpha and beta diversity comparisons. Alpha diversity indices, including richness estimators [Abundance-based Coverage Estimator (ACE), the Chao1 richness estimator, and Observed OTUs], evenness (equitability), species diversity (Shannon–Wiener diversity index and Simpson’s index) and phylogenetic estimators [phylogenetic diversity (PD) whole tree], as well as beta diversity metrics, including weighted UniFrac, unweighted UniFrac, and Bray–Curtis distances, were calculated to compare the microbial communities based on their diversity and phylogenetic structure. Principal coordinate analysis (PCoA) of the beta diversity distances was performed in QIIME. The abundances of OTUs in the samples were then standard normalized, and the microbial taxa (phylum, class, order, family, and genus) were classified using the Rhea pipeline before statistical comparisons (Lagkouvardos et al., 2017). The community function was predicted from the OTU table using Phylogenetic Investigation of Communities by Reconstruction of Unobserved States (PICRUSt, http://huttenhower.sph.harvard.edu/galaxy) with the Kyoto Encyclopedia of Genes and Genomes (KEGG) database (Kanehisa et al., 2008; Langille et al., 2013).

### Statistical Analysis

The data were further analyzed using the following statistical methods. Clinical and demographic data were compared using analysis of variance (ANOVA) or the nonparametric Kruskal–Wallis rank-sum test depending on the normality of the data. Differences in diversity, taxonomic composition, and community function were evaluated using the Kruskal–Wallis or Wilcoxon rank-sum test. PCoA was performed as indicated, with significance of the clustering determined by the nonparametric Adonis test with 9,999 permutations. Correlation analysis within taxa and between taxa and KEGG pathways/blood cell counts were calculated using the Rhea pipeline, which performs a centered log-ratio transformation to eliminate compositionality constraints in taxonomic variables prior to the calculation of Pearson’s correlation (Lagkouvardos et al., 2017). Indicator genera specific to a given group were identified based on the normalized abundances of genera using the R package indicspecies, and the significant indicator value (IV) index was calculated by the 9,999-permutation test (De Caceres et al., 2010). Hierarchical clustering was performed using the R package pvclust with the ward.D2 correlation distance measure, and the *P*-value for clustering was calculated by multiscale bootstrap resampling with 9,999 permutations (Suzuki and Shimodaira, 2006). Statistical analyses and plots were performed using QIIME or R (ver. 3.3.3). The correlation network was visualized using Cytoscape (ver. 3.5.1) (Cline et al., 2007). *P* < 0.05 was considered statistically significant.

### Data Availability

The raw sequencing data from this study are available in the Sequence Read Archive^1^ under accession number SRP120263.

## Results

To explore the oral microbial features of IBD patients, we performed 16S rRNA gene sequencing of 104 salivary samples from 92 individuals (54 patients with active or remissive UC, 13 patients with active or remissive CD, and 25 HCs). The subjects’ demographic and clinical characteristics are summarized in **Supplementary Table S1**, which shows that there were no significant differences in age, gender or BMI among the UC, CD, and HC groups. After pre-processing of the sequencing data, we obtained 5,794,219 high-quality sequences (Phred ≥ Q30) with an average of 55,714 per sample, yielding 1,511 OTUs at a 97% identity cut-off and 1,215 ultimate OTUs without singletons among all the salivary samples (**Supplementary Table S2**).

### Global Phylogenetic Alterations of the IBD Microbial Community

**FIGURE 1:**
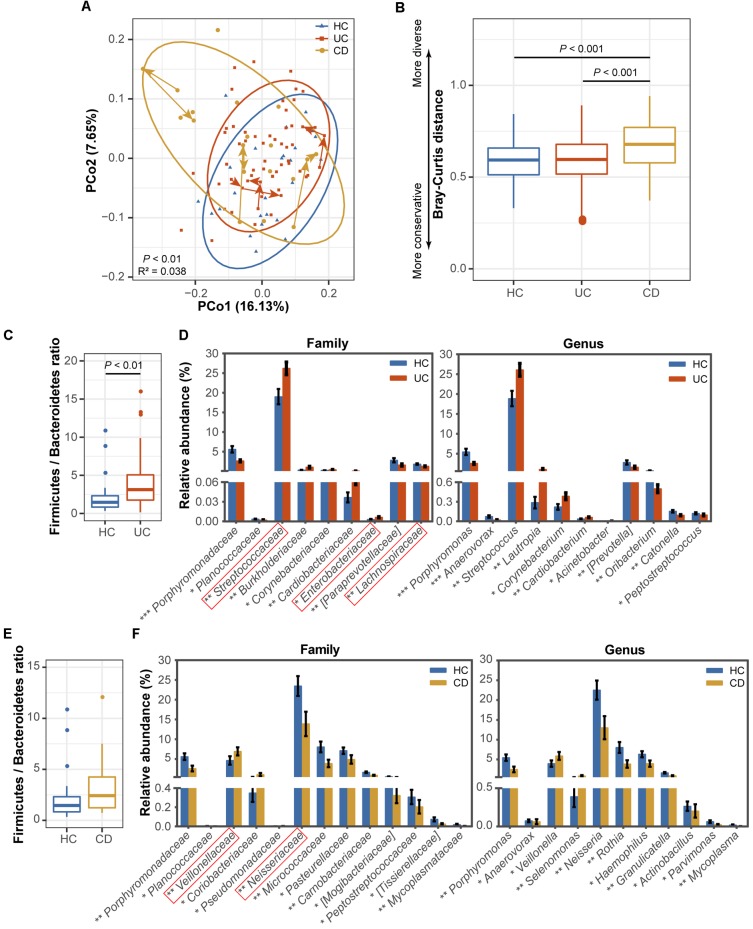
Comparisons of the phylogenetic structure and composition among the ulcerative colitis (UC, 54 subjects), Crohn’s disease (CD, 13 subjects) and healthy control (HC, 25 subjects) microbial communities. **(A)** Principal coordinate analysis (PCoA) based on unweighted UniFrac distances is shown for the UC (red) and CD (yellow) patients and healthy controls (blue). Ellipses represent a 95% confidence interval (CI) around the cluster centroid. Temporal changes from baseline to 1 and 2 months after baseline in the samples obtained from the 3 UC and 3 CD patients with active diseases are indicated by arrows. The 10,664 sequences were rarefied randomly for every sample before calculating pairwise distances. Statistical significance was examined using the Adonis method [nonparametric multivariate analysis of variance (MANOVA)] with 9,999 permutations. **(B)** A boxplot of the Bray-Curtis dissimilarity distance suggesting the degree of community conservation was performed within the three groups. *P*-values were calculated using 9,999 Monte Carlo permutations. **(C)** Comparisons of the Firmicutes/Bacteroidetes ratio and **(D)** taxonomic profiles at the family and genus levels between the UC and healthy populations are shown as a boxplot [median and interquartile range with whiskers) and bar plots (mean ± standard error of the mean (s.e.m.)], respectively. Red boxes in the bar plot emphasize the taxa in this study with the same variation tendencies in the gut (gut information was summarized from previous classical studies as available in **Supplementary Table S4**). The Wilcoxon rank-sum test was used for statistical comparisons. Significance is indicated by ^∗^ (^∗∗∗^ indicates *P* < 0.001, ^∗∗^ indicates *P* < 0.01, and ^∗^ indicates *P* < 0.05). **(E)** Comparisons of the Firmicutes/Bacteroidetes ratio and **(F)** family and genus profiles between CD patients and controls.

To characterize dysbiosis in the oral microbial communities of IBD patients compared to healthy individuals, we first analyzed the alpha (within-sample) and beta (between-sample) diversities of the microbiota to evaluate their overall compositional richness and structural features. In accordance with Said et al.’s (2014) study, no differences were observed in salivary bacterial richness among the UC, CD and HC groups (**Supplementary Figure S1**). However, PCoA based on unweighted UniFrac distances at the OTU level revealed a statistically significant separation of the three groups (Adonis, *P* < 0.01; **Figure 1A**), suggesting different phylogenetic structures. Additionally, a similarity analysis measured using the Bray–Curtis distances revealed a significantly reduced degree of conservation in the bacterial community structure of CD patients compared to those of UC patients and healthy individuals (*P* < 0.001), but no difference was found between the UC patients and HCs (**Figure 1B**).

Next, we identified the microbial taxa at all taxonomic levels and determined the variation in salivary bacterial composition between patients and controls. We detected a total of 15 phyla, 26 classes, 43 orders, 75 families, and 115 genera; consistent with previous observations, the phyla Firmicutes, Proteobacteria, Bacteroidetes, Actinobacteria, Fusobacteria and TM7 constituted the vast majority of the predominant salivary microbiota (>99% of the overall abundance, **Supplementary Figure S2**). By comparing the ratio of Firmicutes to Bacteroidetes, we observed an increasing tendency in both the UC (*P* < 0.01, **Figure 1C**) and CD (**Figure 1E**) communities compared with those of the HCs, although the increase was not significant in the CD group. At the family level, *Streptococcaceae*, *Burkholderiaceae*, *Corynebacteriaceae*, *Cardiobacteriaceae*, and *Enterobacteriaceae* were significantly enriched in UC patients (**Figure 1D**), and *Veillonellaceae*, *Coriobacteriaceae*, and *Pseudomonadaceae* were enriched in CD patients compared with the HCs (**Figure 1F**). In contrast, several families, namely, *Porphyromonadaceae*, *Planococcaceae*, *[Paraprevotellaceae]*, and *Lachnospiraceae*, were significantly depleted in UC individuals (**Figure 1D**), and *Porphyromonadaceae*, *Planococcaceae*, *Neisseriaceae*, *Micrococcaceae*, *Pasteurellaceae*, *Carnobacteriaceae*, *[Mogibacteriaceae]*, *Peptostreptococcaceae*, *[Tissierellaceae]*, and *Mycoplasmataceae* exhibited lower proportions in the CD group (**Figure 1F**). At the genus level, *Streptococcus*, *Lautropia*, *Corynebacterium*, *Cardiobacterium*, and *Acinetobacter* and *Veillonella* and *Selenomonas* were significantly enriched in UC and CD patients, respectively (**Figures 1D,F**). In contrast, two genera, *Porphyromonas* and *Anaerovorax*, were significantly depleted in both UC and CD patients compared to the HCs; *[Prevotella]*, *Oribacterium*, *Catonella*, and *Peptostreptococcus*, and *Neisseria*, *Rothia*, *Haemophilus*, *Granulicatella*, *Actinobacillus*, *Parvimonas*, and *Mycoplasma* were also significantly depleted in UC and CD patients, respectively (**Figures 1D,F**). Collectively, our data confirmed the compositional dysbiosis in UC- and CD-associated oral bacterial communities.

### Correlation Networks of IBD-Enriched and HC-Enriched OTUs

**FIGURE 2:**
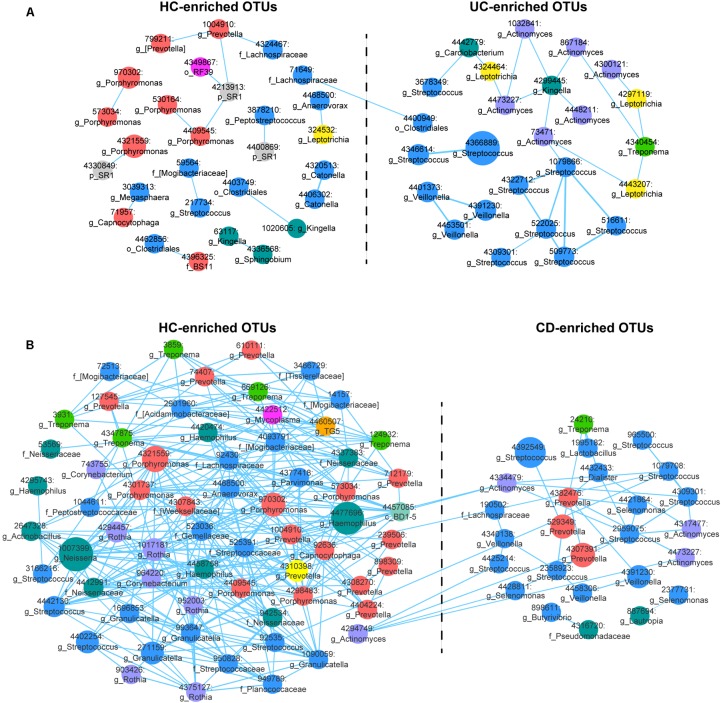
Correlation networks of differentially abundant Operational Taxonomic Units (OTUs) among the UC, CD and HC microbial communities. **(A)** The UC-associated network. Differentially abundant OTUs between the UC and HC groups based on the Wilcoxon rank-sum test were used for network construction. Pearson’s correlation coefficients among the centered log-ratio-transformed OTUs were calculated, and correlations with coefficients ≥ 0.4 and *P*-values < 0.05 were retained. In the network, each node represents an OTU. The node size is proportional to the mean relative abundance of the OTU. Lines between the nodes show positive correlations. **(B)** The CD-associated network.

We then generated abundance-based correlation networks using UC/CD-enriched and HC-enriched OTUs to detect the synergy of taxa across different healthy statuses. Pearson’s correlation coefficients among the centered log-ratio transformed OTUs were calculated, and correlations with coefficients ≥0.4 and *P*-values < 0.05 were retained. For the correlation network between UC- and HC-associated OTUs, 53 nodes and 44 edges were retained (**Figure 2A**). We found that HC-enriched OTUs mainly originated from the phyla Bacteroidetes and Firmicutes, while UC-enriched OTUs were principally from the phyla Firmicutes and Actinobacteria, confirming the higher ratio of Firmicutes to Bacteroidetes and the taxonomic changes in UC. Interestingly, nodes from the genera *Streptococcus*, *Veillonella*, and *Actinomyces* in the UC-enriched group were mainly positively correlated with the within-group nodes from the same genera, respectively, as were *Porphyromonas*, *Prevotella*, and *Catonella* in the HC group. The synergy of OTUs in the same genera enhances the utilization of nutrients, e.g., the co-exploitation of salivary proteins by *Streptococcus*, which is beneficial for their survival in different ecological environments (Van der Hoeven and Camp, 1991; Wickstrom et al., 2009). In contrast, one node from the family *Lachnospiraceae* (71649) in the HC group was positively correlated with a node from the order Clostridiales (4400949) in the UC group (**Figure 2A**). In the CD-related network, we retained correlations consisting of 91 nodes and 367 edges (**Figure 2B**). OTUs originating from the phyla Bacteroidetes, Firmicutes, and Proteobacteria were enriched in the HC group, while other OTUs that were mostly from Firmicutes were enriched in the CD group, validating the evaluated ratio of Firmicutes to Bacteroidetes. Nodes from the genera *Streptococcus, Veillonella*, and *Selenomonas* in the CD-enriched group were mainly positively correlated with each other, as were *Porphyromonas, Prevotella*, and *Rothia* in the HC group. Besides, nodes from *Streptococcus*, and *Actinomyces* in the CD group were positively correlated with nodes from *Streptococcus* and *Actinomyces* in the HCs (**Figure 2B**).

### Involvement of Differentially Abundant Families in Functional Variation

**FIGURE 3:**
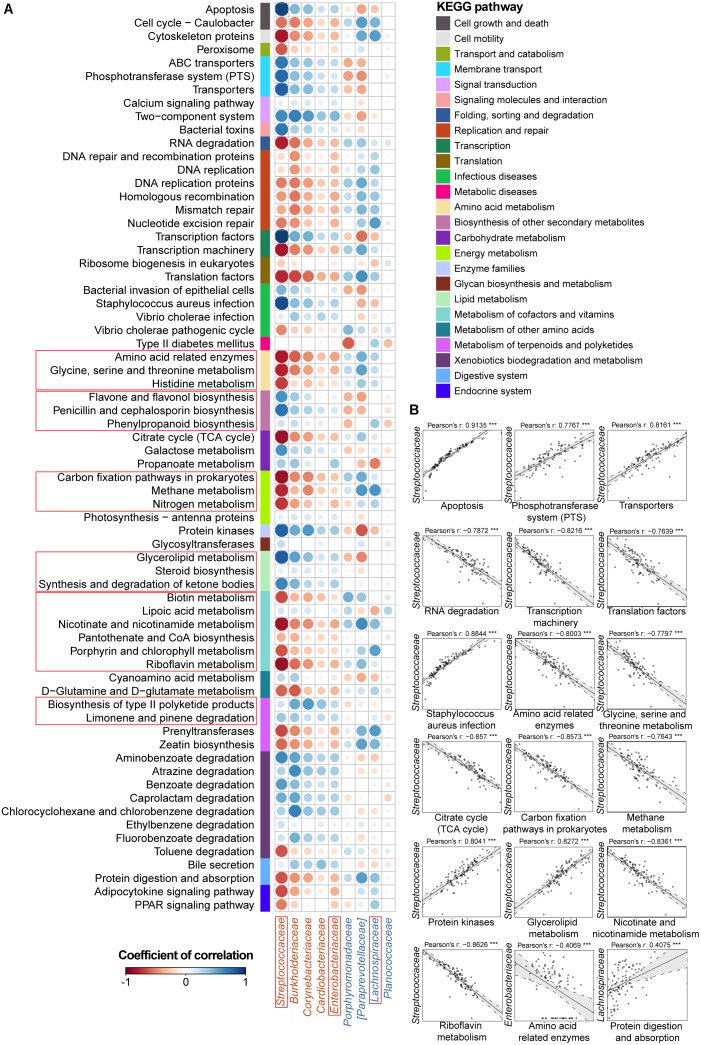
Associations between altered families and functions in the UC microbial community with reference to HCs. **(A)** Pearson’s correlation array between the family and functional variables. Functional pathways were predicted from an OTU table using the PICRUSt algorithm with references from the KEGG database. Differentially abundant highest-detail pathways and families between the UC and HC groups based on the Wilcoxon rank-sum test are listed in rows and columns, respectively. Colored bars adjacent to the highest-detail pathways indicate corresponding upper pathways. The colors of the circles represent the directions of the correlations, and their sizes correspond to the significance of the *P*-values (the larger the circle, the smaller the *P*-value and thus, the higher the significance). **(B)** Individual correlations of *Streptococcaceae*, *Enterobacteriaceae* and *Lachnospiraceae* with correlation coefficients ≥ 0.4 or ≤ -0.4 and their *P*-values are shown (^∗∗∗^ indicates *P* < 0.001, ^∗∗^ indicates *P* < 0.01, and ^∗^ indicates *P* < 0.05). The line in each correlation indicates the fitted linear model, and the gray area within the dashed lines shows the 95% CI for that model.

**FIGURE 4:**
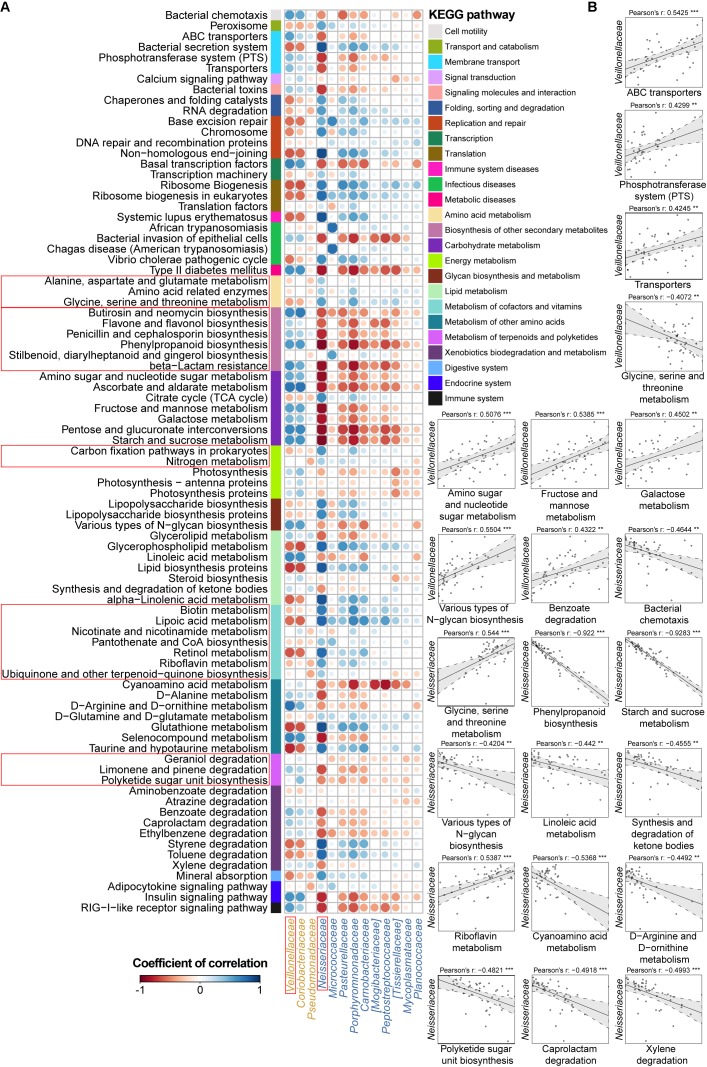
Associations between altered families and functions in the CD microbial community with reference to HCs. **(A)** Pearson’s correlation array between the family and functional variables. Differentially abundant highest-detail pathways and families between the CD and HC groups were calculated using the Wilcoxon rank-sum test. The mid-detail pathways corresponding to differential functions are indicated by colored bars. The colors of the circles in the array represent the directions of the correlations, and their sizes correspond to the significance of the *P*-values (the larger circle represents the smaller *P*-value and thus, the higher significance). **(B)** Individual correlations of *Veillonellaceae* and *Neisseriaceae* with correlation coefficients ≥ 0.4 or ≤-0.4 and their *P*-values are shown (^∗∗∗^ indicates *P* < 0.001, ^∗∗^ indicates *P* < 0.01, and ^∗^ indicates *P* < 0.05).

Another focus of our study was to disclose the functional variation in the IBD salivary microbial community. Therefore, we predicted the microbiota-derived pathways using the PICRUSt algorithm with the KEGG database and compared functional abundances among the UC, CD, and HC groups. The predicted metabolic pathways discriminated UC, CD, and HC individuals in a PCoA analysis, in agreement with our observation for the taxa (Adonis, *P* < 0.01; **Supplementary Figure S4**). In total, we characterized 273 detailed pathways in the present study (odd or obviously unrelated human functions were ignored) (**Supplementary Table S3**). Specifically, the functional changes in UC and CD samples included a loss of genetic information processes (e.g., replication and repair, transcription, and translation) and basic metabolism (e.g., amino acid, energy, cofactor, and vitamin metabolism) (**Figures 3A**, **4A**). In contrast, an increase in the biosynthesis and transport of substances that enhance oxidative stress and virulence (e.g., metabolism of terpenoids, polyketides, carbohydrates and lipids, and biosynthesis of secondary metabolites), bacterial violence (e.g., bacterial toxins and invasion of epithelial cells), enzyme families (e.g., protein kinases), and apoptosis were observed in the UC and CD communities (**Figures 3A**, **4A**). Taken together, the results of the present study demonstrated not only compositional dysbiosis but also functional disturbance.

Therefore, we went a step further to examine correlations between UC-/CD-associated taxa and functional variables to obtain an overview of how specific taxa act during metabolic dysfunction in patient saliva. For UC cases, we characterized positive correlations between the enrichment of *Streptococcaceae* and increased apoptosis, transport, protein kinases, and glycerolipid metabolism as well as negative relationships between *Streptococcaceae* and decreased basic metabolism (amino acid, citrate cycle, energy metabolism, and cofactor and vitamin biosynthesis) (**Figure 3B**). The increase in *Enterobacteriaceae* was negatively related to amino acid-related enzymes (**Figure 3B**). Conversely, the depletion of *Lachnospiraceae* in UC samples was positively correlated with protein digestion and absorption (**Figure 3B**). In CD patients, the enrichment of *Veillonellaceae* had positive correlations with membrane transport, carbohydrate, and glycan metabolism and xenobiotic biodegradation as well as negative associations with glycine, serine, and threonine metabolism (**Figure 4B**). In contrast, the depleted *Neisseriaceae* was related to an increase in the biosynthesis of substances that are beneficial for oxidative stress and virulence (e.g., secondary metabolites, glycans, lipids, terpenoids, and polyketides) and a decrease in transport and glycine, serine, threonine and riboflavin metabolism (**Figure 4B**).

For subjects who had a full set of salivary and blood data (40 UC and 13 CD patients), correlations between the above taxonomic variables and blood cell counts were also analyzed. Interestingly, but reasonably, UC/CD-associated families were all correlated with white blood cells (**Supplementary Figure S5**). Specifically, *Streptococcaceae* and *Lachnospiraceae* in UC samples were related to eosinophils in the blood (**Supplementary Figure S5A**). The enriched *Veillonellaceae* in CD samples were positively associated with leukocytes and lymphocytes, while depleted *Neisseriaceae* were negatively related to lymphocytes, monocytes, and basophils (**Supplementary Figure S5B**). These results demonstrated that dysbiosis of the salivary microbiota could reflect the inflammatory response of the body.

### Microbial Disease Ecotypes Associated With UC and CD

**FIGURE 5:**
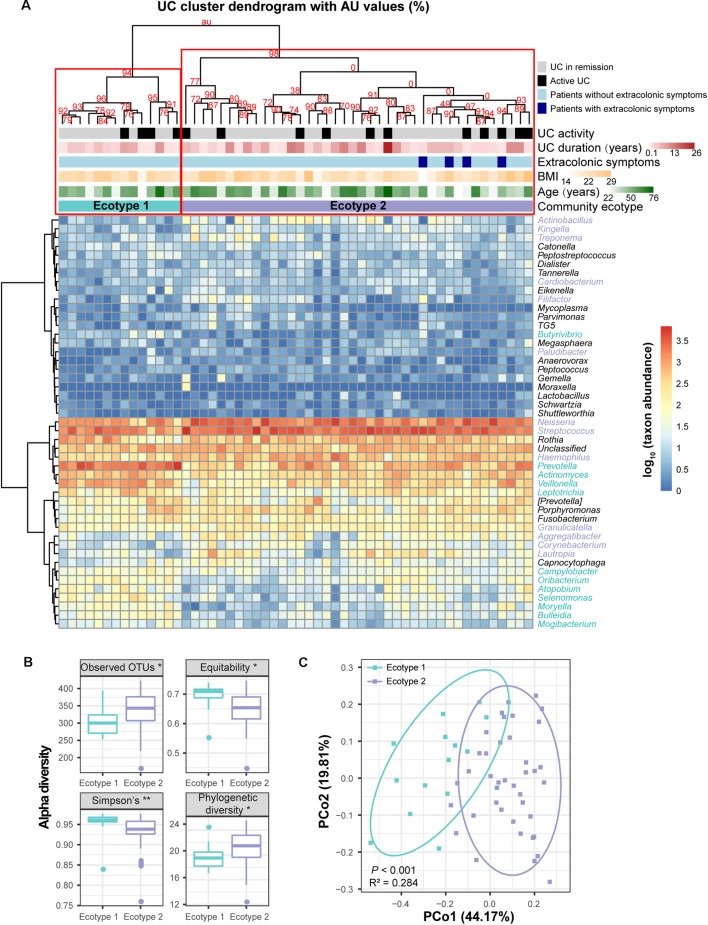
Clustering within the UC microbial community and bacterial diversity of the clusters. **(A)** Hieratical clustering of the UC community (54 baseline samples) with AU values was performed based on the log-transformed genus abundances. Abundant genera with proportions ≥0.01% in the UC microbiota are shown in the column of the heatmap. Two clusters (namely, UC ecotype 1 and ecotype 2) were determined with high (>90%) AU values (the higher the AU value, the greater the significance and thus, the stronger the credibility of the clusters) using multiscale bootstrap resampling with 9,999 permutations. Differentially abundant genera between ecotypes 1 and 2 were calculated by the Wilcoxon rank-sum test, and they are colored turquoise (UC ecotype 1) and lavender (UC ecotype 2) in the column of the heatmap. The clinical (disease activity, durations and extracolonic symptoms) and demographic (age and BMI) features between the UC ecotypes were not significantly different (shown as colored bars at the top of the heatmap). **(B)** Significant alpha diversity indexes between ecotypes 1 and 2 are shown using the Wilcoxon rank-sum test. ^∗∗^ indicates *P* < 0.01, and ^∗^ indicates *P* < 0.05. **(C)** Principal coordinate analysis (PCoA) based on weighted UniFrac distances was performed between ecotypes 1 and 2. *P*-values were calculated using 9,999 Adonis permutations.

To obtain more information about the UC and CD microbial communities, we evaluated whether disease samples could be divided into clusters (hereafter termed ecotypes) using multiscale bootstrap resampling with AU values. Within the 54 UC baseline samples, two robust ecotypes (i.e., UC ecotype 1 and ecotype 2) were determined with high (>90%) AU values (the higher the AU value, the higher the significance and thus the greater the credibility of the clusters) based on the genus-level profile (**Figure 5A**), which were further validated by PCoA (*P* < 0.001, **Figure 5C**). Alpha diversity evaluation revealed that ecotype 1 had a higher biodiversity in its community than ecotype 2 (*P* < 0.01), with less species richness (*P* < 0.05) but more evenness (*P* < 0.05). However, ecotype 1 was less phylogenetically diverse than ecotype 2 (*P* < 0.05), suggesting a set of more distantly related species in ecotype 2 (**Figure 5B**). For the taxonomic divergence, ecotype 1 was mainly enriched with the phyla Firmicutes and Actinobacteria, while ecotype 2 was primarily enriched with Proteobacteria and Firmicutes (**Figure 5A**). Of interest, ecotype 2 exhibited higher proportions of *Streptococcus* and genera from the HACEK bacterial group (*Haemophilus*, *Aggregatibacter*, *Cardiobacterium*, and *Kingella*) (**Figure 5A**). The HACEK group is known to be a team of fastidious gram-negative bacteria that are an unusual cause of infective endocarditis. Although ecotypes did exist across UC patients, they were not driven by demographic (gender, age, and BMI) or clinical (number of patients with active UC, UC duration, and extracolonic symptoms) characteristics in our cohort. Future exploration is needed to obtain information to develop precision medicine strategies for UC individuals.

**FIGURE 6:**
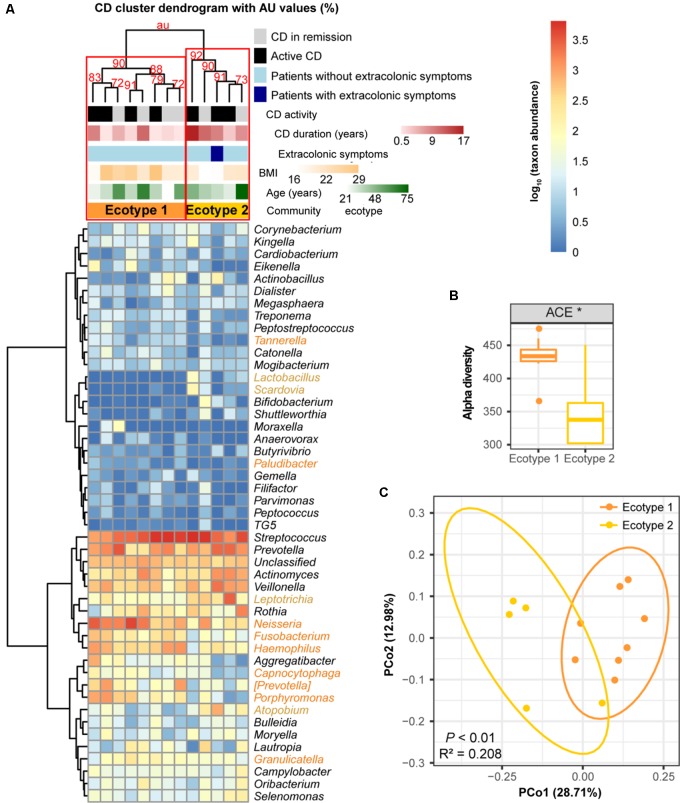
Clustering within the CD microbial community and bacterial diversity of the clusters. **(A)** Hieratical clustering of the CD community (13 baseline samples) with high (>90%) AU values was performed using the same method as described for UC. Two clusters (namely, CD ecotype 1 and ecotype 2) were determined using multiscale bootstrap resampling with 9,999 permutations. The differentially abundant genera between ecotypes 1 (orange) and 2 (yellow) were calculated. CD clinical and demographic features between the ecotypes are shown (no significant differences). **(B)** Significant difference in the alpha diversity index between CD ecotype 1 and 2 are shown. ^∗^ indicates *P* < 0.05. **(C)** Principal coordinate analysis (PCoA) based on unweighted UniFrac distances was performed.

For the 13 CD subjects, we also identified two distinct ecotypes (CD ecotype 1 and ecotype 2) (AU values >90%, **Figures 6A,C**). The community richness was clearly higher in ecotype 1 than ecotype 2 (*P* < 0.05, **Figure 6B**). Enriched genera of ecotype 1 were derived from the phyla Bacteroidetes and Proteobacteria, and those in ecotype 2 were from Firmicutes, Fusobacteria, and Actinobacteria (**Figure 6A**). Similar to the UC population, there was no association between CD ecotypes and demographic or clinical features (**Figure 6A**).

### Genera Indicative of UC and CD Patients

**FIGURE 7:**
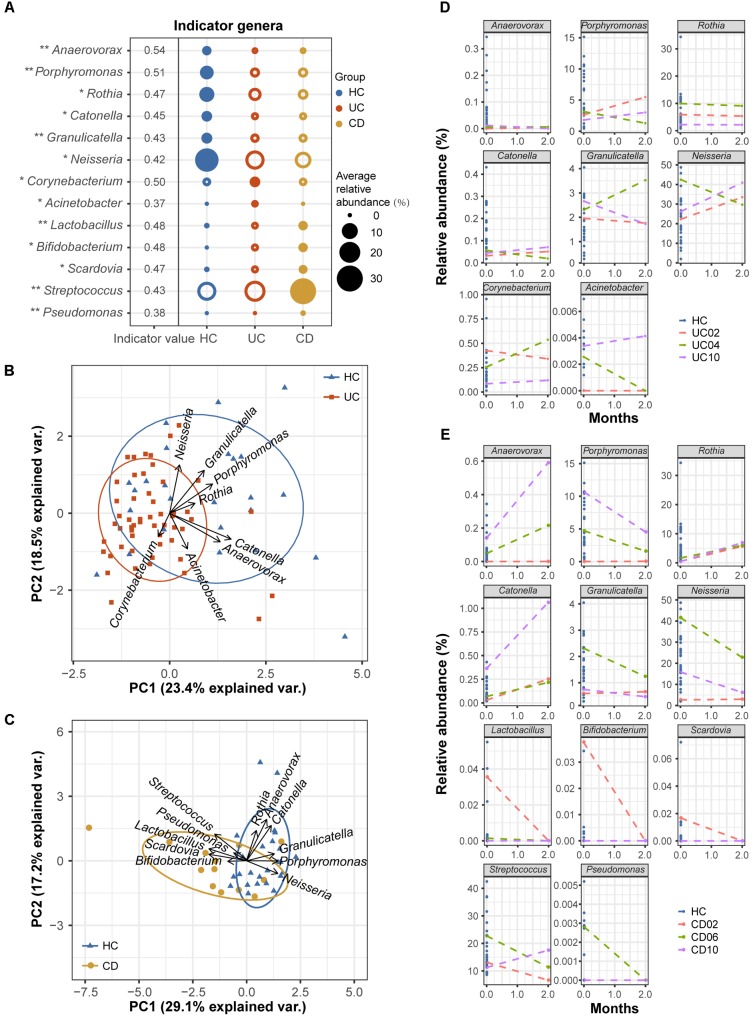
Indicator genera characterizing the UC, CD and HC bacterial communities. **(A)** Indicator species analysis based on the normalized abundances of the genus profile was performed (54 UC, 13 CD, and 25 HC baseline samples). Indicator genera corresponding to each group are designated by solid circles. Corresponding indicator values and their *P*-values are also reported next to the name of the taxon (^∗∗^ indicates *P* < 0.01, and ^∗^ indicates *P* < 0.05). Indicator values (IV) range from zero to one, with larger values denoting stronger indictors. Only indicators with significant *P*-values are shown. **(B)** The separation of UC and HC samples is shown in the PCA biplot using UC- and HC-associated indicator genera. **(C)** The separation of CD patients and HCs is shown using their associated indicator genera. Temporal variation patterns of the above indicators in **(D)** three UC and **(E)** three CD patients with active diseases. The horizontal axis of each plot represents the months from study entry and the vertical axis represents the relative abundances of the indicators. Samples from baseline and after 2 months of standard therapy were used to examine variations. Baseline samples from healthy controls are shown as references.

Although oral microbial dysbiosis in IBD communities was demonstrated compared with those of the HCs, a limited number of taxa indicative of these diseases were known because generalist taxa that occur across diversified groups or rare taxa that are not often sampled are not appropriate indicators. To obtain specific and sensitive oral indicator taxa that are helpful for the long-term screening or monitoring of IBD patients, we performed indicator species analysis using the R package indicspecies based on the genus-level profiles of the baseline samples (54 UC, 13 CD, and 25 HC samples). Considering both relative abundances and relative frequencies, significantly higher indicator values (IV) denote stronger indicators, which could better characterize UC, CD, and healthy statuses. The indicator analysis showed that *Corynebacterium* and *Acinetobacter* were UC-related indicators; *Lactobacillus*, *Bifidobacterium*, *Scardovia*, *Streptococcus*, and *Pseudomonas* were CD-related indicators; and *Anaerovorax*, *Porphyromonas*, *Rothia*, *Catonella*, *Granulicatella*, and *Neisseria* were HC-related indicators (**Figure 7A**). A principal component analysis (PCA) biplot using UC- and HC-associated indicators could distinguish the majority of UC patients from the HCs, as well as the CD and HC groups (**Figures 7B,C**).

To test the robustness of these indicator genera, we examined the temporal variation patterns of the indicator abundances during a 2-month treatment period using a small longitudinal cohort, which included 3 UC and 3 CD patients with active diseases. For the UC cohort, we found that the relative abundances of the UC- and HC-associated indicators for the 2-month treatment samples from patients UC02 and UC10 (red and purple lines) closely approached those of healthy samples compared to their respective baseline samples (**Figure 7D**). However, the indicator abundances of the 2-month sample from patient UC04 (green line) varied less regularly (**Figure 7D**). For the CD cohort, we observed that after 2 months of therapy, the relative abundances of the associated indicators gradually approached those of the HCs (**Figure 7E**). These observations suggested good performance of the oral bacterial indicators.

## Discussion

Aberrant immune function activated by the convergence of genetic, environmental, and microbial elements leads to the pathogenesis of IBD (Kaser et al., 2010). Our results confirmed that the oral microbiota is disturbed during the onset of UC and CD, apart from the well-known gut dysbiosis, and we identified key components of the phylogenetic and functional changes that occur with this disease status (**Figures 1–4**). The longitudinal cases showed microbial migrations along with the recovery of clinical symptoms (**Figure 1A** and **Supplementary Figure S4**). Our work also revealed correlations between IBD-associated taxonomic and functional variables as well as correlations between taxa and blood cell counts, which illustrated the involvement of IBD-related taxa in metabolic activity and their role as an indicator of inflammatory status (**Figures 3**, **4** and **Supplementary Figure S5**). Furthermore, within the UC and CD communities, ecotypes could be identified with strong credibility (AU values >90%). We examined the ecotypes that were not relevant to demographic and disease characteristics (**Figures 5**, **6**). We also further characterized the indicators specific to the diseases, which were validated in the longitudinal cohort (**Figure 7**). To the best of our knowledge, this report is a pioneering study that identified not only the predicted function of the salivary microbiota in human IBD patients but also defined oral microbial ecotypes within individuals with this disease. In addition, we adopted a tailored approach when characterizing indicator taxa of IBD. Altogether, this study broadened our understanding of the oral microbiome associated with IBD patients.

In patients with immune-mediated inflammatory diseases, including IBD, rheumatoid arthritis, and spondyloarthritis, the gut microbiota can exert a profound effect on the host immune system, both locally and globally, changing the human internal environment from an anti-inflammatory to a pro-inflammatory state (Yeoh et al., 2013). Bacteria present in the oral cavity can also be altered by immune dysregulation. In turn, the modified oral microbiota can react against the systemic immune system, resulting in immune disorder intensification as well as the elevated carriage of both activated immune cells and antigenic substances to the gastrointestinal tract. Therefore, oral bacteria could participate in the pathogenesis and development of IBD. Previous research has found that oral *Streptococcus* is able to invade the bloodstream and aggravate colitis induced by enhanced secretion of interferon-γ (Kojima et al., 2012, 2014); oral *Campylobacter* might be involved in the chronic inflammation associated with IBD by inducing the production of pro-inflammatory cytokines from epithelial cells, monocytes, and macrophages (Man et al., 2010); oral *Klebsiella* species could colonize a dysbiotic gut lumen and activate T helper 1 cells, leading to intestinal inflammation (Atarashi et al., 2017; Cao, 2017). However, several recent studies also detected *Helicobacter pylori* in the oral cavity (Cai et al., 2014; Yee, 2016) and showed its potential protective role on IBD by exerting an immunomodulatory action on the intestinal mucosa (Owyang et al., 2012; Wu et al., 2015; Castano-Rodriguez et al., 2017). Therefore, this study targeted the oral microbiome in IBD patients to acquire a better understanding of the oral microbial risk and its role in IBD.

Several recently published studies have indicated that the oral and gut microbiota are – to some extent – correlated with each other during disease states. A metagenomic sequencing study showed that, in the gut microbiota of liver cirrhosis patients, more than half of the patient-enriched, taxonomically assigned species were of oral origin, suggesting their invasion of the gut from the mouth (Qin et al., 2014). Another study using the same sequencing technique reported concordance between the gut and oral microbiomes in individuals with rheumatoid arthritis, highlighting an overlap in the abundance and function of species in different body sites (Zhang et al., 2015). In the case of IBD, we sought to determine whether there is concordance between the gut and oral microbiomes. By comparing our data to classic gut research (**Supplementary Table S4**), surprisingly, we observed that changes in several oral microorganisms in this study were consistent with changes in the gut of IBD patients, i.e., enrichment of *Streptococcaceae* (*Streptococcus*) (Mar et al., 2016) and *Enterobacteriaceae* (Mar et al., 2016) in UC and *Veillonellaceae* (*Veillonella*) (Gevers et al., 2014) in CD, along with depletion of Bacteroidetes (Frank et al., 2007), *Lachnospiraceae* (Frank et al., 2007), and *Prevotella* (Mar et al., 2016) in UC. These taxa performed similar functions in the mouth and gut, namely, decreasing genetic information processes and basic metabolism, accompanied by increasing the biosynthesis and transport of substances that are advantageous for oxidative stress and virulence (Morgan et al., 2012; Gevers et al., 2014). However, the phylum Firmicutes and its genus *Clostridia* exhibited higher abundances in the oral cavity (**Supplementary Figure S3**) in contrast to their changes in the gut of IBD individuals (Gophna et al., 2006; Sokol et al., 2009; Walker et al., 2011). Firmicutes form the most predominant portion of the human oral and gut microbiome, most species of which produce endospores that are resistant to dehydration and can survive in extreme or dysbiotic conditions. Due to the enrichment of Firmicutes, the ratio of Firmicutes to Bacteroidetes displayed an increasing tendency in both UC and CD patients in the present study (**Figure 1**). The Firmicutes/Bacteroidetes ratio is known to be of significant relevance for the human gut microbiota status (Ley et al., 2006), and it is often used as a biomarker in connection with human physiology (Bahl et al., 2012). The shift of the ratio in our data indicated an abnormal physiological state in IBD patients.

Other taxa, such as the depletion of *Haemophilus* and *Neisseria* in the present study, were also reported in the other salivary microbial observation of IBD (Said et al., 2014). Certainly, there were a few different taxa compared with the previous three studies that examined the oral bacteria associated with IBD. For example, *Porphyromonas*, *Anaerovorax*, *Lautropia*, *Corynebacterium*, *Cardiobacterium*, *Acinetobacter*, *Oribacterium*, *Catonella*, *Peptostreptococcus*, *Selenomonas*, *Granulicatella*, *Actinobacillus*, *Parvimonas*, and *Mycoplasma* were also found to be significantly differentially abundant either in UC or CD patients in our study. These microorganisms are all opportunistic pathogens that colonize the oral cavity, which can lead to diseases in an immune-deficient population. There are several possible reasons for the inconsistency among our study and the abovementioned reports. First, our study used MiSeq to sequence the 16S rRNA gene V3–V4 region, whereas others utilized 454 pyrosequencing or microbe identification microarrays. The use of a different sequencing platform might result in discrepant outcomes (Fouhy et al., 2016). Second, our study characterized the salivary microbial communities of Chinese IBD adults, while others evaluated the salivary, subgingival, or mucosal microbiota of Japanese or American adults or children. The distinct food consumption patterns, ethnic backgrounds, age, and niches of different populations have been demonstrated to contribute to variation in the bacterial communities in the oral cavity (Signoretto et al., 2006; Mason et al., 2013; Xu et al., 2015).

Since subjects suffering from IBD usually constitute a heterogeneous population with various environmental, demographic, and genetic features, uneven clinical manifestations, and diverse therapeutic interventions, great inter-subject microbial variability likely exists within individuals with these diseases. Indeed, Mar et al. (2016) demonstrated the presence of four distinct gut microbial community states in a UC cohort. However, the oral bacterial community types within IBD remained unclear. In the present study, we demonstrated that both UC and CD patients could be divided into sub-communities depending on their oral microbial profiles. The sub-communities harbored discrepant species diversity that was not driven by patient demographic and severity characteristics. Research on community ecotypes of these diseases may potentially offer new insight into personalized treatment plans and fitted therapies against the same clinical entity.

Given the reality of oral microbial dysbiosis and the relevance of the oral and gut microbiomes in IBD status, the oral microbiota could play an important role in clinical practice for screening and monitoring IBD patients. The use of oral samples has two major advantages. Oral samples can be quickly and non-invasively collected, contributing to greater patient cooperation as well as lower financial and time burdens for the screening of at-risk individuals. Furthermore, the oral microbiota has been shown to change at a lower rate than the gut microbiota over time (Costello et al., 2009), suggesting a stable diagnosis using oral bacterial markers. Consequently, we conducted indicator species analysis in this study. We found several indicators, i.e., *Corynebacterium*, *Acinetobacter*, *Lactobacillus*, *Bifidobacterium*, *Scardovia*, *Streptococcus*, *Pseudomonas*, *Anaerovorax*, *Porphyromonas*, *Rothia*, *Catonella*, *Granulicatella*, and *Neisseria*, which clearly distinguished different disease and health states.

In summary, this study demonstrated the structural, compositional, and functional dysbiosis of the oral microbiome in patients with IBD and identified disease-related taxa and their relationships with metabolic activity and complete blood counts. Furthermore, within the disease communities, robust sub-ecotypes were observed that were not demographic or severity-specific. We additionally identified genera indicative of the diseases, which were confirmed in a longitudinal cohort. Herein, we should note the application prospects of oral bacteria in identifying patients at risk for IBD and in monitoring their disease courses. However, the oral microbial community in IBD patients is more complex than expected, and further validation experiments are required to gain a better understanding of their oral microbiome.

## Author Contributions

ZX participated in collecting the samples, performing the experiments, analyzing the data, and drafting the manuscript. QZ participated in performing the DNA extraction and revising the manuscript. FC, NC, and TX participated in designing all the experiments and drafting the manuscript.

## Conflict of Interest Statement

The authors declare that the research was conducted in the absence of any commercial or financial relationships that could be construed as a potential conflict of interest.

## Footnotes

1 http://www.ncbi.nlm.nih.gov/sra/

## Supplementary Material

FIGURE S1 Comparisons of alpha diversity among the UC (54 subjects), CD (13 subjects), and HC (25 subjects) microbial communities.

FIGURE S2 Compositional profiles of each sample at the phylum, class, order, family, and genus levels.

FIGURE S3 Differentially abundant phyla, classes, and orders in the UC and CD microbial communities compared with those of the HCs.

FIGURE S4 Principal coordinate analysis (PCoA) based on the Bray-Curtis dissimilarity distance of pathways among the UC and CD patients and HCs.

FIGURE S5 Associations between family variables and complete blood counts in the UC and CD microbial communities.

TABLE S1 Characteristics of individuals investigated in this study.

TABLE S2 Summary of the analyzed sequence information with samples.

TABLE S3 Differentially abundant KEGG pathways in the UC patients.

TABLE S4 Gut bacterial characteristics in IBD.
