# Supplementary figures and images for: Dysbiosis and Ecotypes of the Salivary Microbiome Associated With Inflammatory Bowel Diseases and the Assistance in Diagnosis of Diseases Using Oral Bacterial Profiles
Source: Front Microbiol. 2018 May 30;9:1136. doi: 10.3389/fmicb.2018.01136 (PMC5988890; doi:10.3389/fmicb.2018.01136)

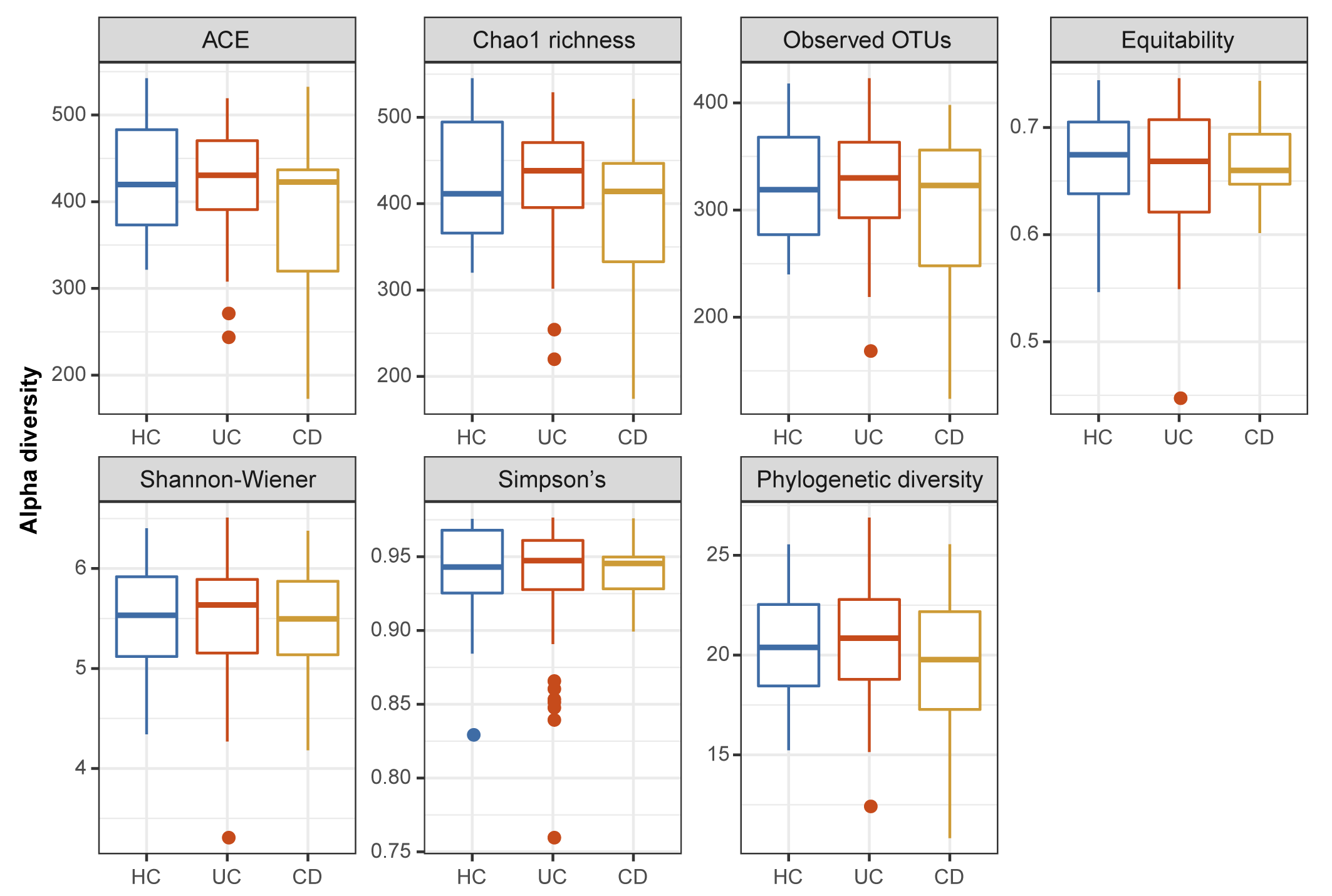

Supplement: FIGURE S1 — Comparisons of alpha diversity among the UC (54 subjects), CD (13 subjects), and HC (25 subjects) microbial communities. [file Image_1.TIF]

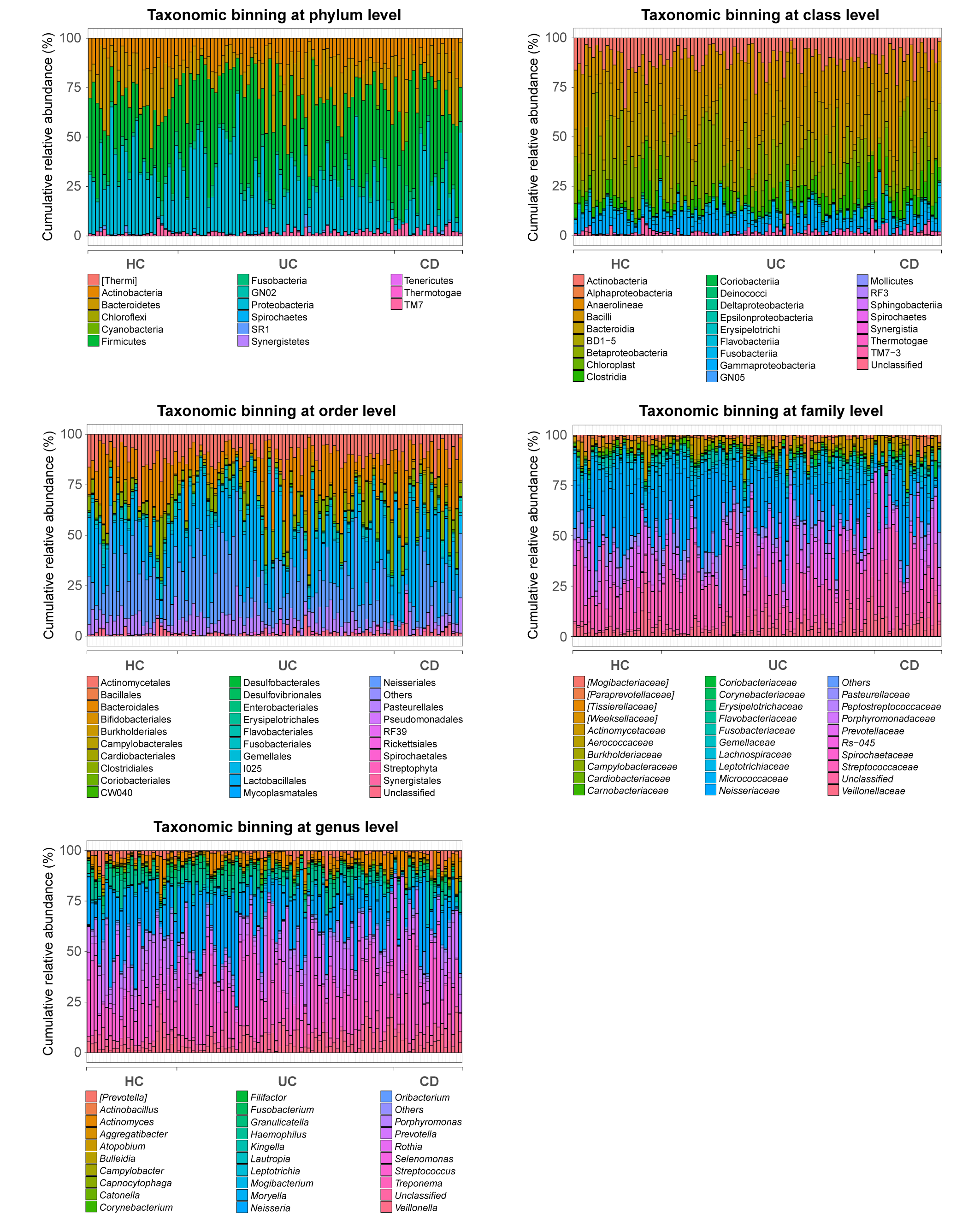

Supplement: FIGURE S2 — Compositional profiles of each sample at the phylum, class, order, family, and genus levels. [file Image_2.TIF]

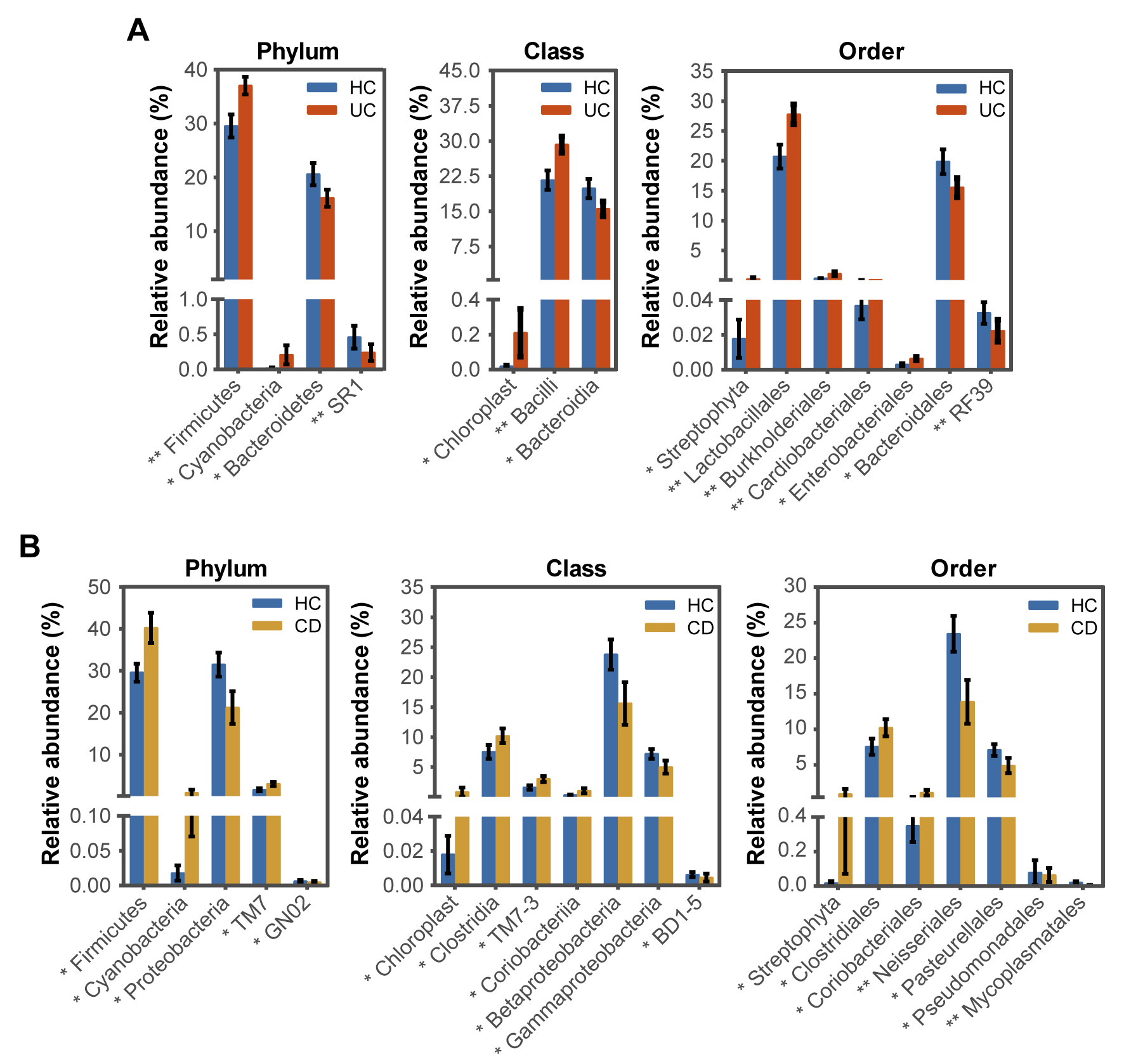

Supplement: FIGURE S3 — Differentially abundant phyla, classes, and orders in the UC and CD microbial communities compared with those of the HCs. [file Image_3.TIF]

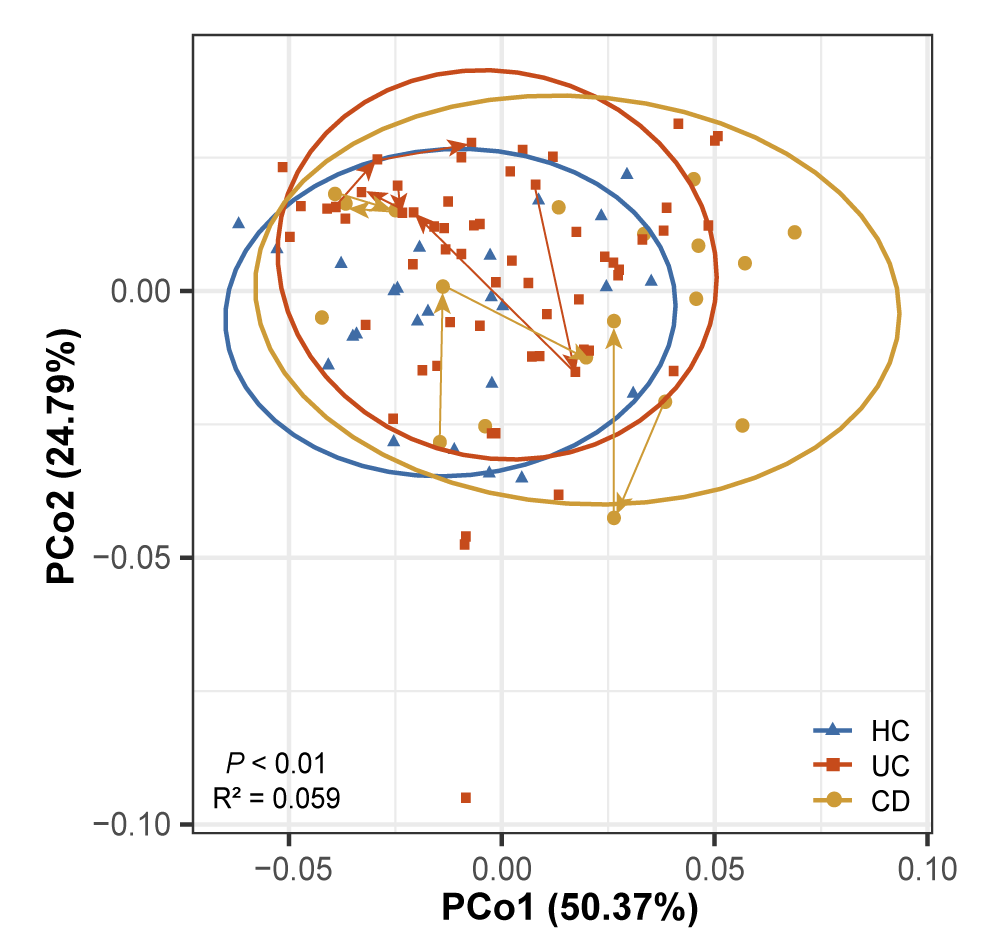

Supplement: FIGURE S4 — Principal coordinate analysis (PCoA) based on the Bray-Curtis dissimilarity distance of pathways among the UC and CD patients and HCs. [file Image_4.TIF]

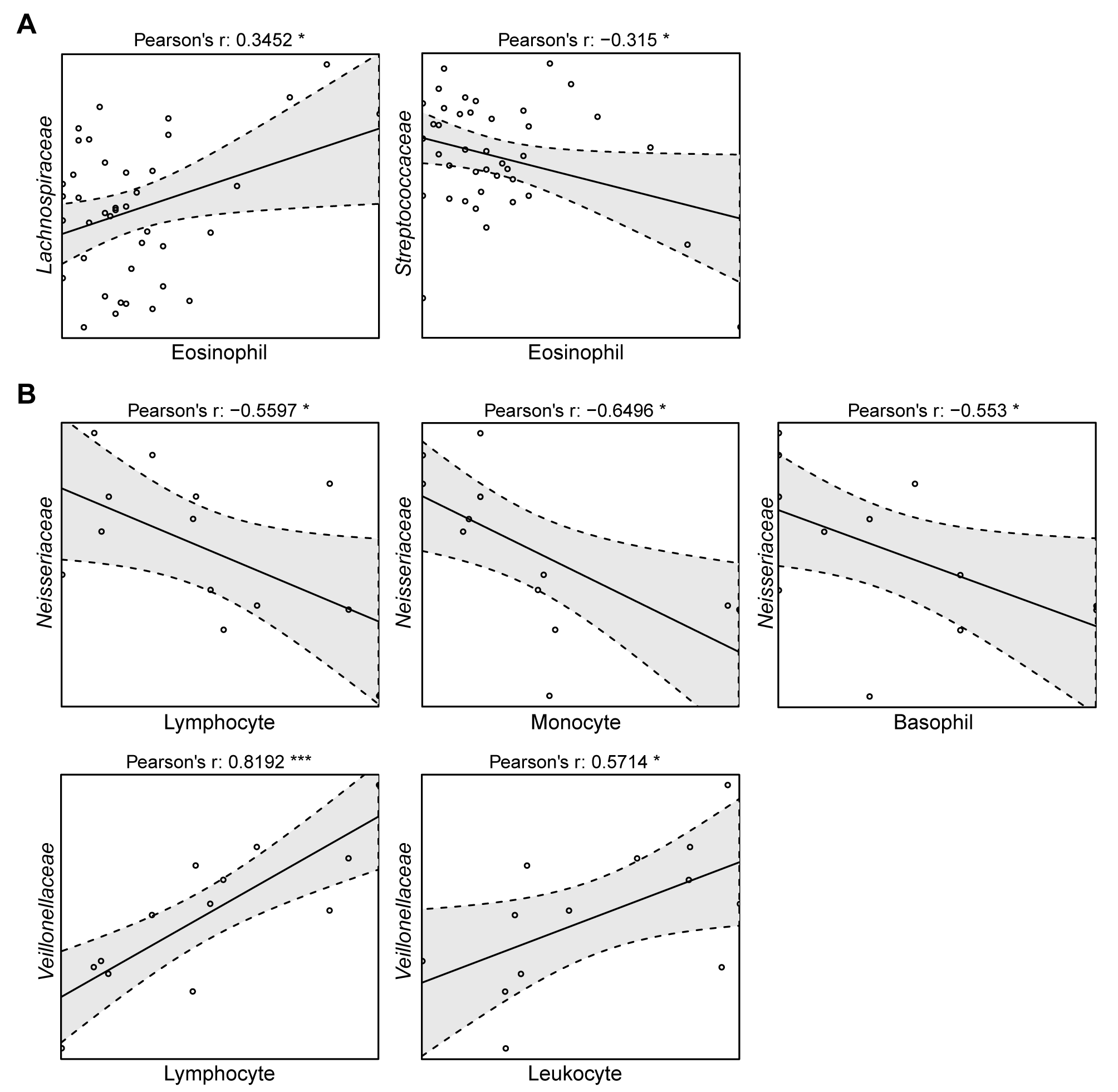

Supplement: FIGURE S5 — Associations between family variables and complete blood counts in the UC and CD microbial communities. [file Image_5.TIF]
